# Anomalous dynamics of water at the octopeptide lanreotide surface

**DOI:** 10.1039/d0ra06237e

**Published:** 2020-09-14

**Authors:** Florian Pinzan, Franck Artzner, Aziz Ghoufi

**Affiliations:** a Institut de Physique de Rennes, UMR CNRS 6251, Université Rennes 1 263 Avenue du Général Leclerc 35042 Rennes France aziz.ghoufi@univ-rennes1.fr

## Abstract

This work reports the study of water dynamics close to the cyclic octapeptide lanreotide from atomistic simulations of hydrated lanreotide, a cyclic octapeptide. Calculation of the hydrogen bonds between water molecules allows mapping of the hydrophilic regions of lanreotide. Whereas a super-diffusivity of the interfacial water molecules is established, a slowdown in rotational dynamics is observed, involving a decoupling between both processes. Acceleration in translation dynamics is connected to the hopping process between hydrophilic zones. Microscopically, this is correlated with the weakness of the interfacial hydrogen bonding network due to a hydrophobic interface at the origin of the interfacial sliding of water molecules. Heterogeneous rotational dynamics of water molecules close the lanreotide surface is evidenced and connected to heterogeneous hydration.

## Abstract 2

Molecular dynamics simulations of a hydrated mutated lanreotide, a cyclic octapeptide, were carried out to characterize its hydration state. We studied the water dynamics close to the peptide using atomistic simulations.

## Graphical abstract

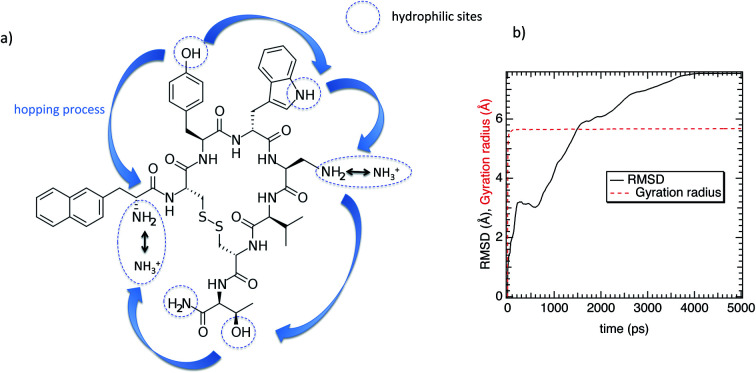

## 1 Introduction

It is now well established that molecular self-assembly is ruled by weak interactions such hydrogen bonds, hydrophobic effects, electrostatic and van der Waals interactions or π–π interactions.^1–5^ However, the molecular mechanisms governing biostructures such as membranes, peptide nanotubes,^1,6–8^ amyloid-related diseases or actin filaments need still to be elucidated. It is then fundamental to gain knowledge of the molecular mechanisms ruling self-assembly processes in order to control them.^6–9^ Much research has thus been devoted to the mechanisms of molecular self-assembly. Peptides have been extensively studied given their simplicity and their versatility to identify specific interactions.^2,10–12^ It has been established that molecular self-assembly is connected to the geometrical complementarity between chemical functions and to the van der Waals non-covalent interactions.

Molecular self-assembly is mainly controlled by an enthalpic contribution related to the strength of the interactions between monomers, *i.e.* electrostatic and van der Waals interactions.^13,14^ The entropic contribution must also be considered to get a full thermodynamic picture of self-assembly. The first entropic contribution is the loss of degrees of freedom of the monomer during self-assembly, contributing to unfavorable energetics.^15,16^ Another factor is the dehydration of monomers during self-association, involving a positive entropic contribution. Therefore, the role of interfacial water, *i.e.* the water molecules located in the first and second hydration shells of the monomer, is crucial in the self-assembly process.^17–19^ To quantify this hydrated state, a local vision of the interfacial hydration of the monomer is necessary. Many experimental and numerical studies have thus been devoted to the hydration of biological molecules such as peptides. It has been shown that the layer of water molecules surrounding a molecule such as DNA plays an important role in both preserving the structure and ensuring its proper biochemical function.^20,21^ The hydration layer further protects the structure; moreover, it is involved both in processing the recognition and binding of restriction enzymes, and in DNA–ligand interactions including protein binding.^22^ Such processes require significant displacement and rearrangement of the water molecules surrounding DNA. A description at the atomic scale of interfacial hydration can then provide insight into the still open and important issue of association, where water-related interactions could dominate the thermodynamic signature of the formation of a dimer.

Among materials that self-organize, the cyclic octapeptide lanreotide shows spectacular assembly because it self-associates into nanotubes in the presence of water.^23^ Lanreotide is a synthetic therapeutic peptide used mainly in the treatment of acromegaly.^6,8,23^ Recently, the peptide packing in these self-assemblies has been determined^9,23^ and numerous levels of organization have been determined; from lowest to highest: (1) dimers of peptides, (2) amyloid filaments generated by the packing of peptide dimers, (3) nanotubes generated by the lateral packing of 26 filaments, and (4) the packing of nanotubes in a hexagonal lattice. All levels of packing organization are controlled by the formation of the dimer, which is the primitive brick ruled by the electrostatic and van der Waals interactions between two monomers. The hydration level of the monomer also plays an important role in self-assembly. Deep knowledge of the hydration of the monomer is therefore necessary to understand and control the highest level of organization. Up to now, experimental studies have been focussed on peptide–peptide interactions to elucidate the molecular mechanism controlling self-assembly, while the hydration state of lanreotide was never explored.

**Fig. 1:**
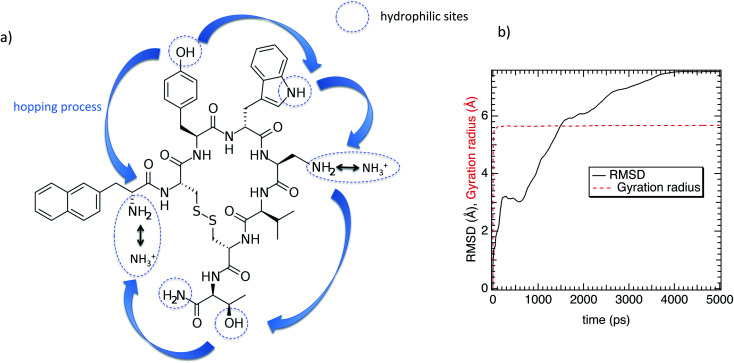
(a) Chemical structure of the lanreotide analogue (l-diaminopropionic acid). (b) Gyration radius and the RMSD as a function of time.

In this work we investigate the interfacial hydration level of a mutated lanreotide (l-diaminopropionic acid) [M-Lanr], described in Fig. 1a, by using molecular dynamics simulations, a relevant tool to explore the structure and dynamics of hydrated materials at the nanoscale.^24–28^ Local properties were computed to dynamically and structurally characterize the interfacial hydration state of M-Lanr. The outline of this paper is as follows. In Section 2, we present the potentials and computational details. The results and discussions are provided in Section 3. The main conclusions of this work are summarized in Section 4.

## 2 Computational details

Positively charged M-Lanr (with total charge +2e) involves the presence of two anions to achieve electro-neutrality. In line with experiments, we opted for the acetate ion as a counterion.^9,23^ The AMBER^29^ model was used to build the force fields of M-Lanr and the acetate ion. In a recent study Man *et al.* showed that the AMBER force field could predict the structural properties of peptides in good agreement with experiments.^30^ Fluitt and de Pablo also identified the AMBERff99SB, AMBERff99SB*, and OPLS-AA force fields to be most suitable for studies of folding and aggregation of polypeptides.^31^ Water molecules were modeled by the rigid, non-polarizable TIP4P/2005 force field.^32^ This water model was considered as a robust model to quantitatively predict the physical properties, taking into account the hydrogen bonding network and accurately modeling the hydration of ions and small solutes.^33–36^ The intramolecular and intermolecular contributions were considered. The intramolecular potential is based on stretching, angle and dihedral potentials, while the intermolecular interactions were modeled from (i) the electrostatic forces between partial charges by means of the Ewald sum and (ii) the Lennard-Jones potential, allowing us to take into account the van der Waals interactions. Partial charges were calculated using the CHELPG approach (CHarges from ELectrostatic Potentials using a Grid-based method)^37^ from first-principles calculations using the 6-31G(d,p) Gaussian-type basis set. Let us note that lanreotide and the acetate ion were modeled by using the original AMBER force field and only the partial charges were re-calculated. The structure of M-Lanr was taken from ref. 38 from XRD measurements. Hydrogen atoms were added and the structure was optimized from the quantum calculation in line with the charge calculation.

The electrostatic interactions were truncated at 12 Å and were calculated using the Ewald sum with a precision of 10^−6^, a convergence of 0.26506 and *k*^max^_*x*_ = *k*^max^_*y*_ = *k*^max^_*z*_ = 38. Short range interactions were modeled by the Lennard-Jones potential using a cutoff of 12 Å. The simulation box was cubic and periodic boundary conditions were applied in the three directions. MD simulations were performed in the *NpT* statistical ensemble, such that *N* is the number of particles, *T* is the temperature and *p* is the pressure. Molecular dynamics simulations were performed at *T* = 300 K and *p* = 1 bar using a time step of 0.001 ps to sample 10 ns (acquisition & equilibration phases). All MD simulations were carried out using the DL_POLY package^39^ using a combination of the Velocity Verlet and the Nose–Hoover thermostat and barostat algorithms^40,41^ with relaxation times of 0.1 ps and 0.5 ps, respectively. The initial configuration was built by a random distribution of water in the presence of M-Lanr and acetate ions. To be in line with the experimental water fraction,^9^ 11 000 water molecules were considered in an initial cubic box with length (*L*) 70 Å.

Regarding the internal structure of M-Lanr, the gyration radius and the asphericity were calculated. The shape of the macrocyclic molecule was estimated from the inertia tensor **S**.^42^ Diagonalization of **S** results in three eigenvalues, which sum to the mean-squared radius of gyration 〈*R*_g_^2^〉, and the largest of which corresponds to an eigenvalue vector representing the long axis of the macrocycle. In Fig. 1b we report *R*_g_ as a function of time. As shown in Fig. 1b, *R*_g_ ∼ 5.4 Å with small fluctuations that suggest rigidity of the cavity. The Root Mean Square Displacement (RMSD) profile of M-Lanr was evaluated at the atomic scale to understand the overall stability. The conformational variation and flexibility of a structure is reflected by the root mean square deviation value, where a small fluctuation denotes fewer fluctuations. From Fig. 1b, the RMSD of M-Lanr deviated much more at the initial phase, as the flexible nature of the protein was higher.

## 3 Results and discussion

**Fig. 2:**
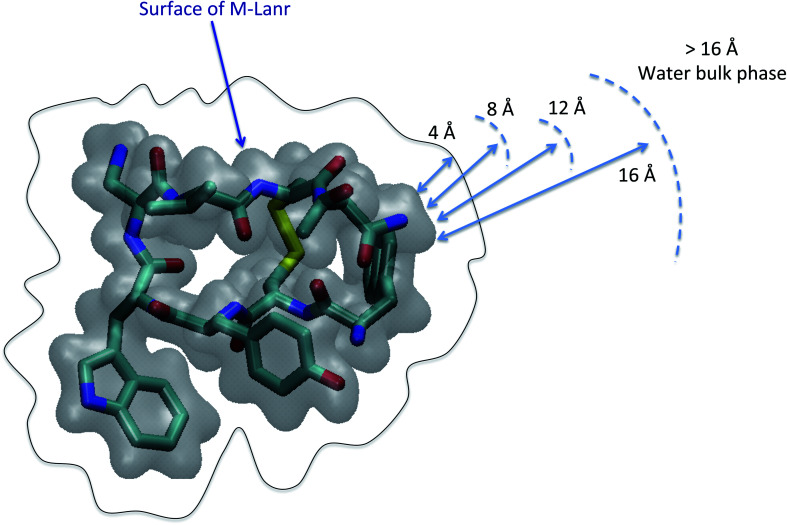
Scheme illustrating decomposition of the shell to calculate the local properties. The Connolly surface of M-Lanr is highlighted in gray. Cyan, yellow, red and blue colors correspond to the carbon, sulfur, oxygen and nitrogen atoms, respectively. For clarity, hydrogen atoms are not considered.

**Fig. 3:**
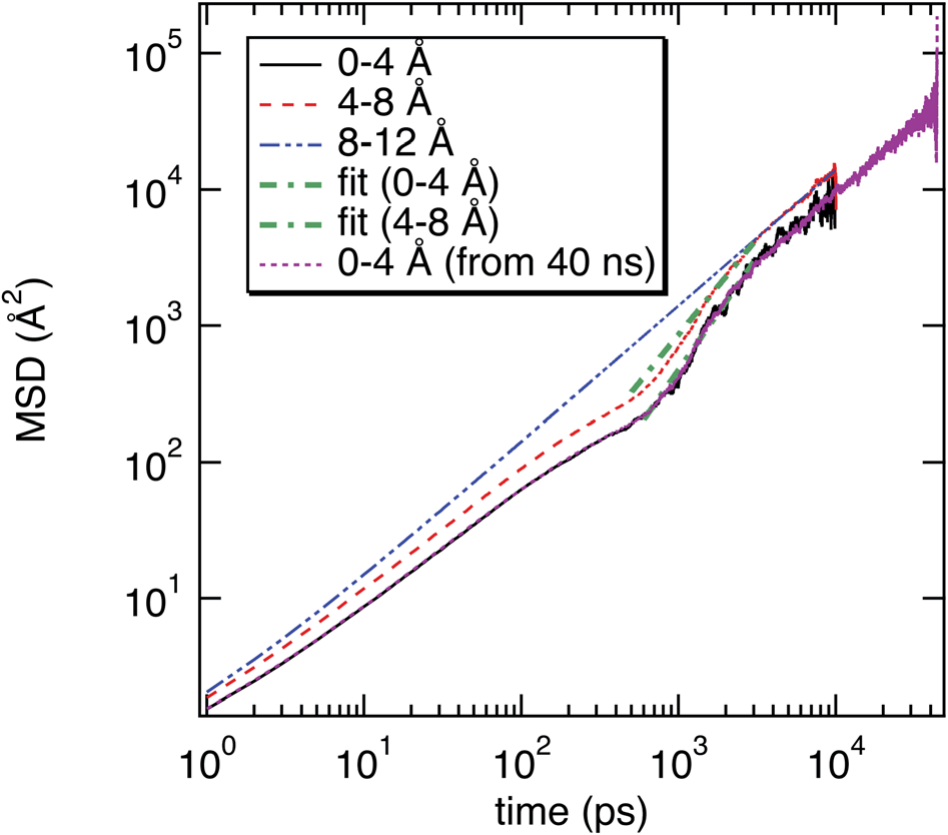
Mean square displacement (MSD) of water molecules as a function of their location with respect to the surface of M-Lanr and as a function of time. Fits of MSD of water molecules using a power law are also provided. For water molecules close to the surface of M-Lanr (between 0–4 Å), the MSD calculated from MD simulation of 40 ns is also reported.

As illustrated in Fig. 2, the local properties were investigated by considering four layers of 4 Å around lanreotide, 0–4 Å, 4–8 Å, 8–12 Å, and 12–16 Å. Beyond 16 Å the bulk phase is recovered. The layers were calculated by considering the distance between each atom of lanreotide and the water molecules. This distance of 4.0 Å corresponds to the first hydration shell of M-Lanr. It was established from the calculation of the radial distribution function between the water molecules and the atoms of M-Lanr. Furthermore, as the radius of a water molecule is around 3 Å, to get a sufficient number of water molecules into the hydration shell we increased the upper limit to 8.0 Å. A distance of 3.0 Å was also considered as the distance of the first hydration shell. Nevertheless, distances smaller than 4.0 Å involved poor statistics, leading to strong fluctuations and noise in the calculations of local properties. The number of hydrogen bonds per water molecule (*n*_HB_) was managed by considering the geometrical criteria established by Chandler and Luzar,^43,44^ such that a hydrogen bond exists if the distance between the hydrogen and oxygen atoms of two molecules is smaller than 2.5 Å and if the distance between oxygen atoms is smaller than 3.5 Å. By considering these two distance criteria, the angle criterion corresponding to an angle between two O–H and O–O vectors smaller than 30° was fulfilled. In the bulk phase the TIP4P/2005 water model provides *n*_HB_ of around 3.91, which is in line with a tetrahedral geometry.^45^ The local *n*_HB_ values of water molecules are reported in Table 1. As shown in Table 1, close to the surface of M-Lanr, *n*_HB_ falls to 3.47, breaking the tetrahedral structure. That is the result of the excluded volume due to the truncation of the hydration shell at the interface. Interestingly, *n*_HB_ increases as water molecules move away from the M-Lanr surface to recover the bulk value, *i.e.* 3.91. A decrease in *n*_HB_ indicates a less cohesive interfacial hydrogen bonding network, which could lead to an increase in the interfacial translational dynamics. Dynamics was then evaluated from the calculation of the mean square displacement (MSD) in the three shells:1
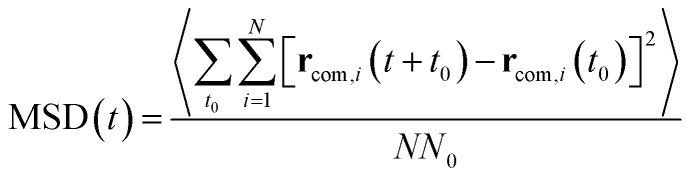
where **r**_com,*i*_ is the position of the centre of mass of molecule *i*, *t*_0_ is the time origin, *N* is the number of molecules and *N*_0_ is the number of *t*_0_. To compute the MSD of confined water in the shell we only considered the molecules that left and entered the shell between two origin times. In Fig. 3 we report the MSD of water molecules in three regions. As exhibited in Fig. 3, the MSD of water molecules are found to be different as a function of their location with respect to the surface of M-Lanr. Furthermore, a time crossover around 0.3 ns is evidenced close to the water/M-Lanr interface. Interestingly, as shown in Fig. 3, this crossover and this change in dynamics are also recovered from MD simulations of 40 ns, which suggests that anomalous dynamics is not time dependent. From the MSD and by using the generalized Einstein relation, the dynamical regime can be determined through the coefficient *α*, such that

**Fig. 4:**
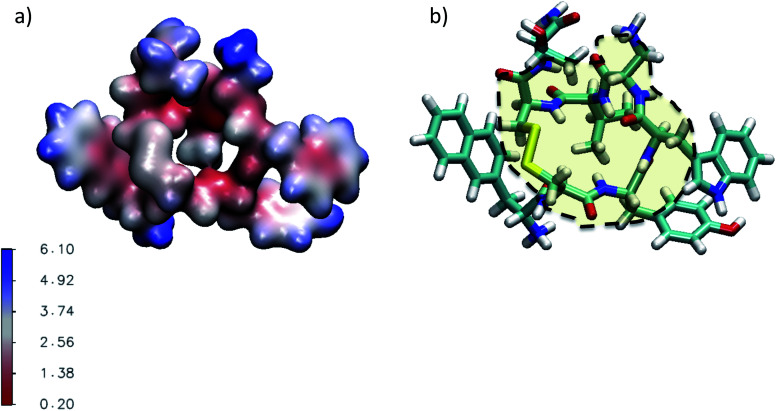
(a) Three dimensional representation of the average water number around the atoms of M-Lanr located at 4 Å. (b) Illustration of the hydrophobic cavity (yellow zone) limited by the dashed line.

2 MSD(*t*) = *Dt*^*α*^,

where *D* is the diffusivity, *α* = 1 corresponds to a diffusive regime, *α* < 1 is connected to a sub-diffusive process, while *α* > 1 is linked to a super-diffusive process. The case of *α* = 2 corresponds to ballistic dynamics.^46–48^ Let us mention that for *α* ≠ 1, the diffusion is considered anomalous. For a diffusive regime the diffusivity is related to the self-diffusion coefficient (*D*_S_) extracted from Einstein’s relation in one dimension (MSD(*t*) = 6*D*_S_*t*). *α* was calculated from a fit of the MSD by using eqn (2). The values of *α* as a function of the water location with respect to the water/M-Lanr interface are reported in Table 1. Close to the interface (0–4 Å and 4–8 Å), *α* was calculated in two regions delimited by a time crossover, between 0 and 0.3 ns (*α*_1_) and between 0.5 and 3 ns (*α*_2_). As shown in Table 1, at a short time *α*_1_ < 1, highlighting a slight sub-diffusive regime, while at a long time *α*_2_ > 1, which is in line with a super-diffusive regime. Interestingly, beyond the water/M-Lanr interface *α*_2_ decreases, whereas *α*_1_ increases to recover a diffusive regime. At the interface an acceleration of water mobility is then observed that could be correlated with the decrease in *n*_HB_ close to the water/M-Lanr surface. Whereas many works have reported a slowdown^17–19^ of the interfacial water mobility, an acceleration of the translational dynamics is observed in the case of M-Lanr. Interestingly, this phenomenon could be compared to the increase in translational mobility of water molecules confined close to a hydrophobic surface such as graphene or nanotube carbon.^28,49^Fig. 4 shows that the 3-dimensional profile of water number around M-Lanr (at a distance < 4 Å) highlights a weakly hydrated surface. This hydrophobicity is borne out by the calculation of *n*_HB_ between M-Lanr and the water molecules, which was found to be close to 0.07 by considering water molecules located at the water/M-Lanr interface. Typically, the decrease in *n*_HB_ between the water molecules and between water and M-Lanr clearly suggests a hydrophobic nature of the M-Lanr surface, leading to the sliding of water molecules along its surface.^28^ Moreover, the decrease in *n*_HB_ per water molecule from 3.91 to 3.47 involves a less cohesive interfacial hydrogen bonding network, leading to an increase in translational degrees of freedom. Eventually, the sub-diffusive regime observed at short times is the result of the confinement effect in the interfacial region.^50^ Indeed, the interfacial water is confined between the M-Lanr surface and the water molecules in the layer beyond 4 Å, leading to a slowdown in the translational mobility given the constricted region at the nanoscale. The decrease in size of the region where the water molecules can diffuse leads to (i) a decrease in translational mobility and (ii) an excluded volume at the origin of the break in HB of the interfacial water molecules. To surpass the sub-diffusive regime, super-diffusion probably must be connected to an additional mechanism to speed up the water molecules.

**Table 1:** Number of hydrogen bonds per water molecule (*n*_HB_), *α*_1_ (value of *α* between 0 and 0.3 ns), *α*_2_ (value of *α* between 0.5 ns and 3 ns), and relaxation time (*τ*_R_) as a function of the location of water molecules with respect to the M-Lanr surface. *α* is the parameter in the generalized Einstein relation, MSD(*t*) = *Dt*^*α*^. For *n*_HB_ the calculation uncertainty is around 0.02

	*n*_HB_	*α*_1_	*α*_2_	*τ*_R_ (ps)
0–4 Å	3.47	0.87	1.99	18.7
4–8 Å	3.71	0.89	1.53	6.9
8–12 Å	3.82	1.0	1.0	5.1
12–16 Å	3.91	1.0	1.0	4.9

**Fig. 5:**
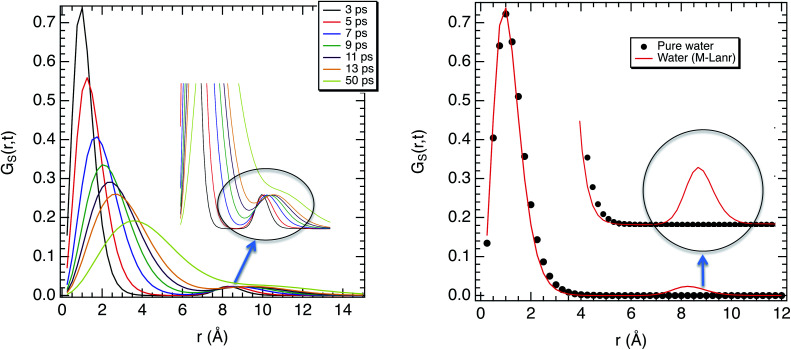
(a) Self van Hove function as a function of the covered distance for different times at 300 K. (b) Self van Hove function as a function of the covered distance at 3 ps for water in solution with M-Lanr and in the pure bulk phase.

The super-diffusivity next to the M-Lanr surface could also be attributed to the heterogeneous structures in hydrogenated sites. Indeed, Fig. 1a shows different hydrogenated sites probably involved in the hopping process.^51^ Possible translational jumps can be evidenced by computing the self van Hove function (*G*_S_(**r**,*t*)), 3
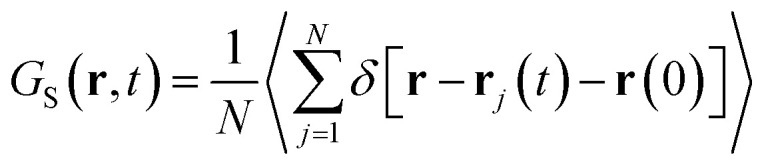
where **r**_*j*_(*t*) is the position of the centre of mass of the water molecules at time *t*, and *N* is the number of water molecules. In Fig. 5a we report *G*_S_(**r**,*t*) from 3 ps to 50 ps. Fig. 5a shows that at short times (between 3 ps and 5 ps) the main peak remains at a distance less than 1 Å from 3 to 50 ps, which indicates small displacements. Furthermore, in Fig. 5a the main maximum of the van Hove function is progressively shifted toward larger distances, which is in accordance with processes observed in the bulk water phase. However, the distributions are non-Gaussian and the second peak expands between 7 and 9 Å, corresponding to the translational jumps and the hopping process^35^ that could be related to the Lévy flight.^46^ To verify this result, *G*_S_(**r**,*t*) of water in a pure bulk phase was calculated. Fig. 5b shows that no hopping is observed in the bulk phase, contrary to water in the presence of M-Lanr, which validates our calculation.

Fluctuations in HB number close to the interface can also be the origin of local anomalous diffusion. However, the standard deviation of *n*_HB_ was found to be close to 0.02 with small fluctuations, in line with a hydrophobic surface stabilizing the structure.^52^ Indeed, the Root Mean Square Deviation (RMSD) between the structures obtained at *t* and the conformation at *t* = 0 was found to be tiny, close to 0.3 Å, suggesting a strong degree of rigidity of M-Lanr, probably due to the hydrophobic surface inducing a kind of folding. Furthermore, the “breathing” ability of the hydrophobic cavity has been evaluated by calculating the time evolution of its gyration radius. An average distance of 5.4 Å with a standard deviation of 0.7 Å was found, leading to evidence of low flexibility and weak structural fluctuations of the hydrophobic cavity.

**Fig. 6:**
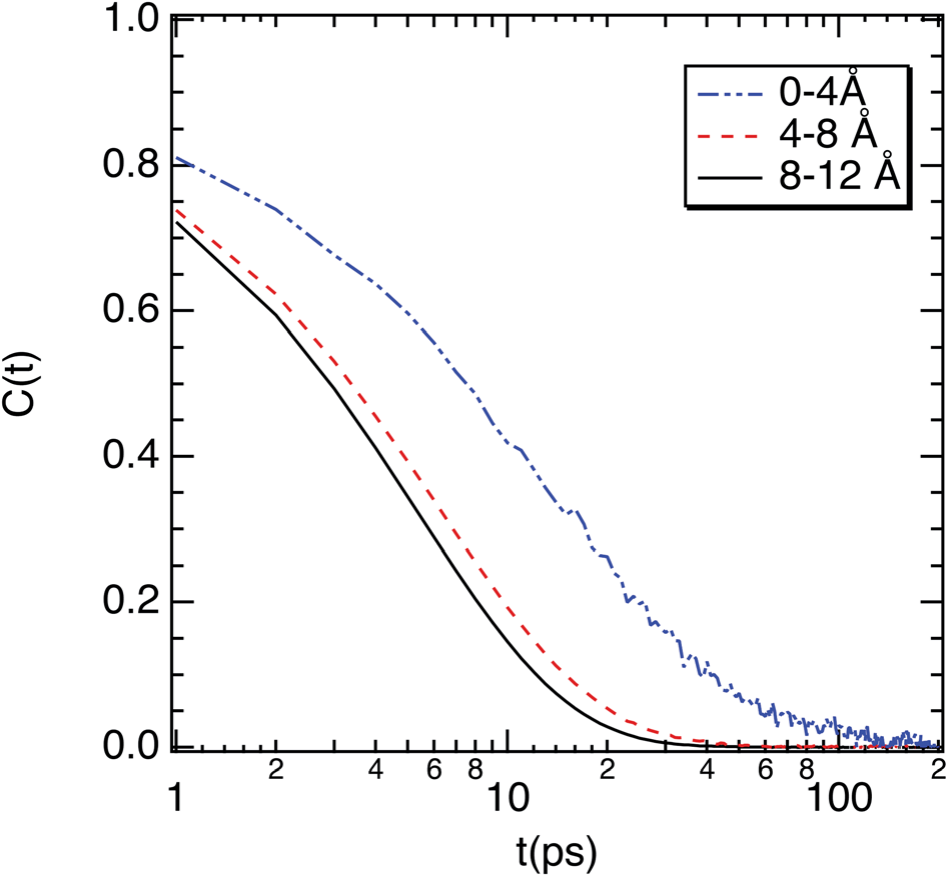
Correlation function (*C*(*t*)) of the dipole moment vector of water molecules as a function of their location with respect to the surface of M-Lanr.

To complete our study the rotational dynamics was investigated from the calculation of the relaxation time (*τ*_R_) of water molecules as a function of their location with respect to the M-Lanr surface. *τ*_R_ was computed from 
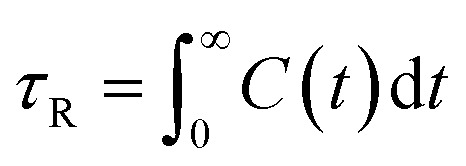
. *C*(*t*) was calculated as4
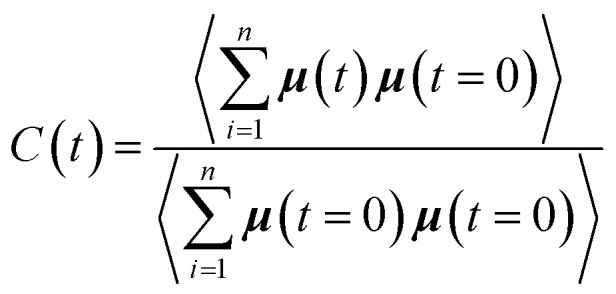
*C*(*t*) of water molecules located in the three local regions are reported in Fig. 6. This figure highlights different decays in *C*(*t*). Indeed, close to the surface (0–4 Å) the decay is much slower, involving a slowdown in the rotational dynamics. The rotational dynamics speeds up as the water molecules move away from the M-Lanr surface to recover the bulk value from 12 Å. Furthermore, Table 1 evidences that the relaxation time increases as the water molecules get closer to the M-Lanr surface, highlighting a slowdown in rotational dynamics leading to a decoupling with the observed increase in the translational dynamics. Table 1 shows a decrease in *n*_HB_ of water molecules next to the interface, involving a decrease in rotational motion in terms of angular jump, a process that takes part in the exchange of HB.^18^ Additionally, Fig. 4 evidenced strong hydrated sites (hydrogen atoms of NH_3_ and OH groups) involving strong anchoring of water molecules close the lanreotide surface. Indeed, the relaxation time of water next to the NH_3_ and OH groups is found to be around 17.9 ps, in line with the value (18.7 ps) reported in Table 1 for all water molecules confined in the interfacial region. This suggests that the high value of *τ*_R_ is the result of water molecules around both NH_3_ and OH groups. Finally, let us mention that the rotational dynamics was found to be similar in both diffusive regimes across the time-crossover observed in Fig. 3; this supports the decoupling of translational and rotational dynamics.

**Fig. 7:**
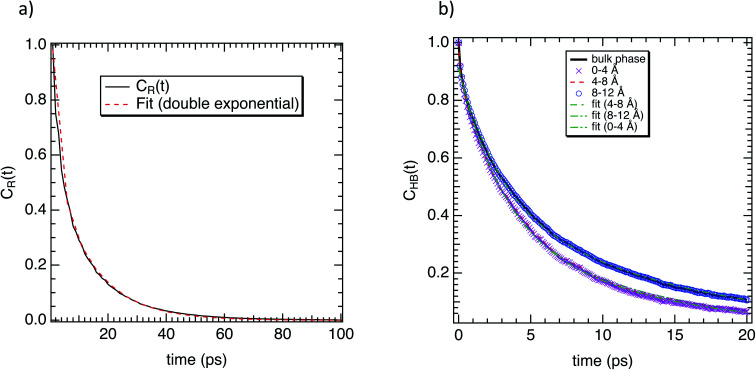
(a) Correlation function (*C*_R_(*t*)) of water molecules near to the surface of M-Lanr. The fit to double exponential decay is also represented. (b) Hydrogen-bond correlation function of water molecules as a function of their location with respect to the surface of M-Lanr.

To complete our dynamical study, we consider the residence time of water molecules located in the interfacial region. The mean residence time is calculated from the correlation function *C*_R_(*t*), defined as5
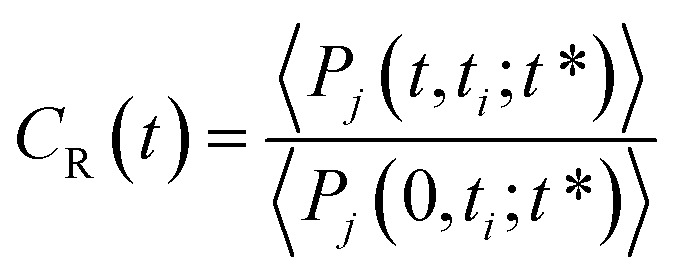
where *P*_*j*_(*t*,*t*_*i*_;t*) is the Heaviside unit step function, which is equal to 1 if the water molecule *j* lies within the interfacial region at both time *t*_*i*_ and *t*_*i*_ + *t*, and in the interim does not leave the interfacial zone for any continuous period longer than t*. Otherwise, the unit step function is equal to zero. This function gives the probability of a water molecule to stay in the interfacial region during time *t*. The parameter t* is introduced to take into account molecules which leave the interface temporarily, but for a time period shorter than t*. The calculations are carried out for a t* value of 2 ps.^53^ Beyond 2 ps the residence time strongly fluctuates. The time correlation function *C*_R_(*t*) can be fitted to second-order exponential decay. The longer time corresponds to the residence time of water molecules, while the shorter time is connected to the time of an escape of the molecule located close to the border of the interface. As shown in Fig. 7a, *C*_R_(*t*) is well adjusted to second-order exponential decay. A residence time of 13.1 ps was found with the same order of magnitude as a relaxation time of 18.7 ps. This highlights that the water molecules are strongly bonded to the octopeptide.

Furthermore, we investigated the hydrogen-bond dynamics in the three shells by introducing the hydrogen-bond correlation function6
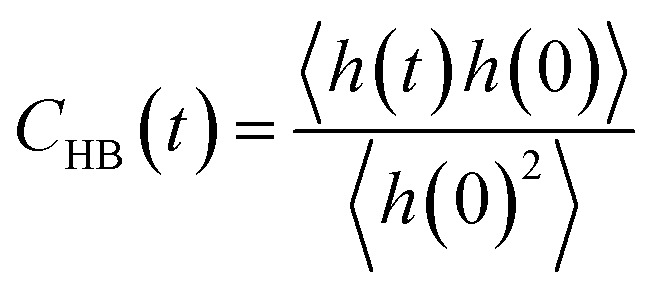
where *h*(*t*) is a hydrogen-bond population descriptor, which is unity when a tagged pair of molecules is hydrogen-bonded at time *t* and is zero otherwise. This correlation function was calculated for all water molecules defined to be hydrogen-bonded at both times 0 and *t*, which describes the probability of the tagged pair of molecules being hydrogen-bonded at time *t* given that the pair was hydrogen-bonded at time 0. The calculated hydrogen-bond correlation function is shown in Fig. 7b, and is best described by a series of two exponential decay functions. Two time constants obtained from the exponential fit are identified as the average hydrogen bond lifetimes, which are 2.7 and 13.3 ps for water near the surface of M-Lanr and in the bulk phase, respectively. This result indicates that the hydrogen-bond dynamics in liquid water occur on either a very fast (breaking and formation of a hydrogen bond) or a slow time scale (collective rearrangement^54^). At the interfacial region (0–4 Å) the hydrogen bond lifetimes are *XX* ps and *YY* ps. The faster time increases, while the slower time decreases with respect to the bulk phase. Gradually, for water molecules to move away from the interfacial region the bulk values progressively recover. At the interface the increase of the faster time could be connected to the decrease in rotational degrees of freedom (see relaxation time) due to the confinement of water molecules in the interfacial region.

## 4 Concluding remarks

In this work, molecular dynamics simulations of a hydrated mutated lanreotide were carried out to dynamically and structurally characterize the hydration state of M-Lanr. Calculation of the number of hydrogen bonds (*n*_HB_) per water molecule allowed us to highlight a decrease in *n*_HB_, leading to weak hydration of M-Lanr and greater mobility of the interfacial water molecules.

The translational dynamics of water was also studied and anomalous dynamics was found close to the water/M-Lanr interface. Indeed, a weak sub-diffusive regime at a short time, followed by super-diffusion, has been established. The sub-diffusive regime is the result of a confinement effect close to the surface, leading to a slowdown in the translational mobility given the constricted region at the nanoscale involving excluded volume and then a breaking of HB of the interfacial water molecules. The super-diffusive process is due to (i) the decrease in *n*_HB_ between the water molecules and between water and M-Lanr (due to the hydrophobic nature of the M-Lanr surface), leading to the sliding of water molecules along its surface. Moreover, the decrease in *n*_HB_ per water molecule involves a less cohesive interfacial hydrogen bonding network, leading to an increase in translational degrees of freedom, and (ii) a hopping process between hydrogenated sites speeds up the translational dynamics.

Eventually, the rotational dynamics was also explored and a decoupling with translational dynamics has been exhibited. Indeed, an increase in the relaxation time of the interfacial water molecules in relation to the water in the bulk phase has been highlighted. That was correlated with a decrease in the exchange of HB between water molecules given the decrease in the interfacial water number and the heterogeneous hydration. This work paves the way for studies of the dimerization process and formation of M-Lanr nanotubes to highlight the role of water in the self-assembly process.

## Conflicts of interest

There are no conflicts to declare.

## Supplementary Material
